# Real wage growth in the U.S. health workforce and the narrowing of the gender pay gap

**DOI:** 10.1186/s12960-021-00647-3

**Published:** 2021-08-28

**Authors:** Janis Barry

**Affiliations:** Department of Economics, Fordham University, 113 West 60th Street, New York, NY 10023 USA

**Keywords:** Recessions and healthcare workers, Gender pay gaps, Healthcare labor markets

## Abstract

**Background:**

Healthcare has been identified as a job engine during recent recessions in the U.S. Whether the healthcare sector provides better than average pay remains a question. This study investigates if wages grew with the expanding demand for healthcare workers between 2001 and 2017. Wage growth in the (1) physicians and surgeons, (2) nurse, (3) healthcare practitioner and technical, (4) healthcare support, and (5) direct patient care jobs are examined. The gender pay gap in each occupation is investigated.

**Methods:**

The American Community Survey (ACS) public use microdata sample (PUMS) for 2001, 2004, 2008, 2013, and 2017 were used to derive hourly wages for full-time, full-year workers aged 18–75. The cumulative percent change in unadjusted, median hourly wages between 2001 and 2017 was calculated for each occupation. Quantile regression estimates predicted a median hourly wage for men and women by year and job after adjusting for differences in demographics, industry, and hours worked.

**Results:**

Unadjusted median wage growth was 9.92% for nurses, 5.68% for healthcare practitioners, and 37.6% for physicians between 2001 and 2017. These rates are roughly above the estimated national rate of wage growth at the 50th wage percentile. In healthcare support and direct patient care occupations, workers experienced either stagnant or negative wage growth. Women had lower occupational wages than men.

**Conclusion:**

The slow or negative median wage growth in all but the physician occupation between 2004–2008 and 2008–2013 confirms that healthcare wages in the U.S. are not recession-proof, unlike healthcare employment. Generally, women's earnings grew at rates that were higher or less negative than rates for men. This trend contributed to narrowing the gender pay gap in every occupation except for nurse.

**Supplementary Information:**

The online version contains supplementary material available at 10.1186/s12960-021-00647-3.

## Introduction

Healthcare has been identified as a job engine during recent recessions in the U.S. This sector is characterized as recession-resistant and capable of generating "good jobs", especially for women with low and middle-skill sets. Whether the healthcare sector provides better than average pay remains a question. Women in healthcare were paid less than men with comparable skills who work in the manufacturing and construction sectors [1]. Many entry-level jobs do not provide a career path, with lower-waged jobs now representing the more substantial proportion of all healthcare employment [2–4].

This study investigates whether wages grew along with the expanding demand for healthcare workers between 2001 and 2017. Real or inflation-adjusted wage growth rates in the (1) physicians and surgeons, (2) nurse, (3) healthcare practitioner and technical, (4) healthcare support, and (5) direct patient care occupations are examined. Workers in these occupations directly interact with patients in delivering healthcare services.

The time frame includes events such as the moderate 2001 recession after the World Trade Center bombing, the rise in unemployment due to the Great Recession beginning in 2007 and continuing into 2010, and the economic recovery years of 2013–2017, during which the Affordable Care Act (ACA) was implemented. Three questions are addressed. First, what were the occupation-specific, real median wage trends for full-time, full-year healthcare workers before, during, and after the Great Recession? Secondly, within occupations, what were the major wage determinants? Thirdly, in which occupations did the gender pay gap narrow after adjusting wages for differences in demographics, industry, and hours worked?

## Background

In 2017, healthcare employment as a percent of total U.S. employment was 11%. The number of full-time, year-round workers increased from 5 to 9 million between 2000 and 2017, and most workers were women [5]. In the U.S. and much of the world, occupational clustering by gender is a distinctive feature of health care employment [6].

Healthcare added jobs during the U.S. recession in 2001 and the financial crisis dated December 2007 to June 2009. Oceania and Canada also recorded increased healthcare employment during the Great Recession. Yet, healthcare employment in Europe fell during this time [7]. Austerity measures during the economic crisis caused many European countries to cut salaries and fees for doctors and nurses [8]. However, in the U.S., the average healthcare wage between 2001 and 2014 increased, but remained lower than the national average [9].

The scale of private sector funding and provision of healthcare services gives U.S. employers significant leverage in determining wage rates across healthcare industries. Alternatively, many European countries have national, centralized wage-setting procedures that develop from collective bargaining agreements with trade unions that significantly impact pay in public hospitals and other settings [8]. Therefore, comparing healthcare wages in the U.S. with those found in other OECD countries is difficult, although, during recessionary periods, employment conditions across countries exhibit similarities [7].

In the U.S., the Great Recession encouraged economic restructuring and a reorganization of healthcare industries. As the economy recovered, many workers were moved from higher-paying, often unionized hospital jobs to work in lower-waged ambulatory and long-term care facilities [10, 11]. Hospital consolidation increased employer bargaining power and contributed to slower wage growth for nurses and pharmacists [12]. In 2010, the ACA was passed, and the expansion of health insurance and a rapidly growing older population effectively increased healthcare service demand [2].

Dramatic employment growth in the healthcare sector is associated with rising labor costs. Economists alarmed with increasing healthcare expenditures caution that physicians are overpaid [13]. Some suggest that healthcare employment should be rolled back, layoffs enacted, and remaining jobs given to those who would work them at lower cost [14]. Earnings for physicians and nurses are indeed higher than for comparable workers in the non-healthcare sector of the economy [15, 16]. Yet, even if the government were to lower its payments to the highest earners in the health system (e.g., physicians and surgeons), the effect on national healthcare spending would be minimal [17]. Correspondingly, reducing nurses' earnings would also have a limited impact.

### Professionalism and gender in healthcare

The division of labor in healthcare reinforces professional boundaries with occupational closure strategies that can limit the earnings of those with only vocational qualifications [10, 15]. Cross-nationally, occupational hierarchies distinguish jobs requiring significant educational training, credentials, and licensing (high status) from those having fewer employment preconditions (low-status) [18, 19]. Higher wages for professionals reflect the derived demand for healthcare services and the greater bargaining power and status associated with their occupation. While increased requirements can raise salaries for highly experienced registered nurses, such credentials do little for improving the pay of certified nursing assistants [11]. During 2005–2015, wage inequality increased between professional and non-professional workers in healthcare [10]. And certifications and other closure strategies did not benefit women's wages as they had for men [1].

Disparities in earnings among men and women employed in the same healthcare occupations are not unique to the U.S. Between 2006 and 2014, across lower and upper-middle-income countries, the gender wage gap in healthcare increased while remaining relatively constant in higher-income countries [6]. Gender and health system hierarchies are embedded with institutional practices attached to social characteristics such as race, ethnicity, class, or caste. Fair and accurate professional productivity evaluations are hidden in compensation policies, allowing bias to determine standards and who meets them [19].

Cross-national explanations for the gender wage gap in healthcare include women’s concentration in specific jobs, specialty choices in medicine, labor market discrimination, the devaluation of caring skills in pay structures, and depressed bargaining power among women [20–23]. Care work by female nurses is often regarded as less valuable than the medical interventions performed by predominantly male physicians [24]. Furthermore, immigrant women and women of color overwhelmingly inhabit the lower-waged care jobs in the U.S. and lack regulatory protections or union representation [1, 3, 25]. Consequently, in the highest-skilled physician and nurse occupations and non-professional healthcare support jobs, pay gaps by gender, race, and nativity are evident [3, 10, 20, 21].

### Data and statistical analyses

The American Community Survey (ACS) public use microdata sample (PUMS) for 2001, 2004, 2008, 2013, and 2017 were used to derive hourly wages. Five years of ACS sample data were concatenated, and five datasets were created for each occupational group. The ACS is the largest, individual-level data set capable of tracking healthcare occupations in the U.S. [26]. Conducted annually by the U.S. Census Bureau, it reaches roughly 295,000 U.S. addresses per month. Households are required by law to respond, and response rates are generally about 95%. The 2001 and 2004 ACS had approximately 1.1 to 1.2 million observations per year. By 2017, this increased to nearly 3.5 million individuals. ACS statistics uniquely provide estimates of consistently defined demographic and economic variables.

Occupations were identified using the Census Bureau's 2010 ACS classification scheme [26]. This scheme organizes homogeneous occupations into clusters. Examples of jobs found within each category and their relevant Census code follow physician and surgeon (3060: (hospitalists, urologists)); nurse (3000–3540: (registered nurse, nurse practitioners); other healthcare practitioner and technical (3000–3540: (chiropractors, dentists)), and healthcare support (3600 -3650: (orderlies, dental assistants)). A fifth category was compiled to include jobs not coded as healthcare occupations in the ACS [27]. This cluster is not homogeneous. The first six jobs demand specific qualifications, while the remaining two have minimal educational or licensing requirements. Direct patient care jobs include medical/health services managers (0350), social/community service managers (0420), psychologists (1820), social workers (2010), counselors (2000), various community and social services specialists (2020), medical/dental/ophthalmic laboratory technicians (8760) and personal/home care aides (4610).

A median hourly wage was determined for all full-time (30 or more hours a week), full-year (51 weeks) workers aged 18–75, or for physicians and surgeons, subset to aged 35 years and older. The ACS wage value was divided by usual hours and weeks worked. Median wage growth rates by occupation were calculated. Then, using a quantile regression model, the natural logarithm of the CPI-adjusted, trimmed hourly wage was adjusted for demographic, industry, and hours worked differences. Results from the 50th percentile of the hourly wage distribution are reported.

Independent variables included in the regression analyses were gender (female/male), age (18–34, 35–40, 41–49, 50–59, 60–75), race/ethnicity (non-Hispanic Black/African-American, Hispanic/Latino, non-Hispanic White, Asians/others), nativity (native-born, naturalized citizen, non-citizen), education (high school or lower, some college, Bachelor's degree or higher), and hours of work (30–34, 35–40, 41–50, 51 or more). Four healthcare industries, including hospitals, ambulatory care (physicians' offices, outpatient care centers), and long-term care (home healthcare, nursing care facilities), were included. The fourth industry consists of "other" locations where healthcare employees reported they worked. Largely comprising the government and education sectors, it also included community/social service organizations and retail pharmacies. The listed variables do not capture everything that could affect median hourly wages, so the state of residence and fixed-year effects were included to control for observed and unobserved influences.

The multivariable regression analyses predicted the log median hourly wage for each occupation using weighted, pooled cross-sections of the ACS so that results are nationally representative. Quantile regression modeling was performed using the R computer language. Robust standard errors for the estimated intercept and coefficients were made using the Huber Sandwich estimator [28].

### Descriptive statistics

Additional file 1: Table S1 provides the pooled-sample characteristics by year for the sample used in the wage regression analyses. For each of the explanatory variables, percentages by gender are also reported. The data confirms the health workforce has grown, and occupational shares have changed over time. Healthcare support’s share declined between 2001 (19%) and 2017 (16.8%). Nurses increased their share between 2013 (20.7%) and 2017 (21.6%) after relative flatness between 2001 and 2013. Direct patient care generally trended upward, reaching 28.9% of the health workforce in 2017. Healthcare practitioners experienced a decrease in share from 2001 to 2013, followed by a substantial increase from 27.5% to 28.9% between 2013 and 2017. The proportion of physicians and surgeons generally trended downward, constituting 5.5% of the total workforce in 2001 but only 5.2% by 2017.

Healthcare support and physicians were the only two occupations that lost workforce share between 2001 and 2017. Declines in the physician share are attributed to retirements, whereas low pay and high turnover in healthcare support increase the difficulty of recruitment to this occupation [3, 29]. All other occupational shares expanded due to rapid growth in healthcare demand [5, 9, 16].

Women were 74.1% of the workforce in 2001, and this increased to 76.5% by 2017. Their representation rose in direct patient care, healthcare practitioners, and physicians between 2001 and 2017. The percentage of non-Hispanic Whites working in healthcare declined, as did the number of native-born workers. Latinos and Asians, along with naturalized citizens, grew their workforce share, while Black workers saw their employment share decline after 2008, only to rebound by 2017.

Workers aged 50–59 and 60 years or older increased their share between 2001 and 2017, as did workers aged 18–34. The percentage of workers with higher education credentials increased significantly. Women exhibited higher levels of educational attainment by 2017. The proportion of the health workforce located in the four healthcare industries was relatively stable. However, women saw job declines in hospitals, while their share of employment in ambulatory and the "other" categories grew between 2001 and 2017. The percentage of female workers reporting working 35 to 40 or 41 to 50 h a week increased between 2001 and 2017. Among all workers, the percentage working 51 h or more declined.

### Median wage results

Table 1 reveals that wage growth rates in all occupations were highest between 2001 and 2004. From 2004 to 2008, healthcare support, direct patient care, and healthcare practitioner workers experienced negative wage growth. Nurses and physicians also registered slower wage growth, with physicians having a rate of 1.3% compared to their 2.47% rate for 2001–2004. By 2008–2013 healthcare support workers continued to have negative wage growth, and nurses saw real wage declines. Direct patient care and healthcare practitioners experienced less than 1% annualized wage growth between 2008 and 2013, while physicians realized an improved rate of 2.16%. Between 2013 and 2017, workers in direct patient care and healthcare practitioner jobs suffered wage declines during this period of economic recovery. Healthcare support and nurses had growth of less than 1%. In contrast, physicians saw wages grow at 2.45%.

**Table 1:** Median wages by year, occupation with annualized percentage change in real wages

Occupation	2001	Annualized	2004	Annualized	2008	Annualized	2013	Annualized	2017	Cumulative
		2001–2004		2004–2008		2008–2013		2013–2017		2001–2017
		%△ wage		%△ wage		%△ wage		%△ wage		%△ wage
Healthcare support	$13.61	1.02	$14.03	−0.18	$13.93	−0.72	$13.43	0.56	$13.73	0.88
Nurses	$29.73	2.41	$31.88	0.45	$32.45	−0.26	$32.03	0.51	$32.68	9.92
Direct patient care	$21.77	0.81	$22.30	−2	$20.51	0.16	$20.67	−0.88	$19.94	−8.4
Healthcare practitioners	$22.68	2.48	$24.37	−0.67	$23.72	0.59	$24.42	−0.47	$23.97	5.68
Physicians/surgeons	$59.36	2.47	$63.77	1.32	$67.14	2.16	$74.40	2.45	$81.70	37.6

Sample is full-time/full-year workers, aged 18–75, except physicians and surgeons are ≥ 35 years of age

Source: American Community Survey, 2001, 2004, 2008, 2013, 2017

Overall, between 2001 and 2017, professional workers registered the fastest cumulative median wage growth. Specifically, physicians' wages grew almost four times faster than nurses' (37.6% versus 9.92%) and over six times faster than the rate of 5.68% in healthcare practitioner occupations. Paraprofessional workers in healthcare support had a cumulative wage growth rate of less than one percent. In contrast, the mixed professional direct patient care category experienced a negative wage growth rate of −8.4%.

### Regression-adjusted median wage results

Table 2 reports the conditional quantile regression coefficients taken at the 0.50 percentile of the wage distribution. The gender dummy coefficients in Table 2 are estimates of the occupational wage penalty for women in 2001, after adjustments for the other covariates. Being a woman decreased the median wage in 2001 (the reference year) by approximately (e^−.1452^)–1 × 100) 16% in healthcare support, by 4% in Nursing, 13% in direct patient care, 15% in healthcare practitioners, and 23% in the physician surgeon category.

**Table 2:** Quantile regression estimated coefficients

	Healthcare support occupations	Nurses	Direct patient care occupations	Healthcare practitioner and technical occupations	Physicians and surgeons (35 and older)
Intercept	2.9095 (0.0330)***	3.5219 (0.0153)***	3.6710 (0.0201)***	3.5866 (0.0192)***	4.3472 (0.0287)***
Sex					
Female	−0.1452 (.0278)***	−0.0436 (0.0118)***	−0.1369 (0.0111)***	−0.1664 (0.0141)***	−0.2555 (0.0318)***
Age					
18 to 34	−0.1708 (0.0064)***	−0.2602 (0.0043)***	−0.2644 (0.0064)***	−0.2465 (0.0063)***	N/A
35 to 40	−0.0282 (0.0071)***	−0.1027 (0.0048)***	−0.0467 (0.0070)***	−0.0429 (0.0069)***	−0.0087 (0.0111)
41 to 49	0.0014 (0.0068)	−0.0517 (0.0043)***	0.0081 (0.0067)	−0.0094 (0.0064)	0.0460 (0.0085)***
50 to 59	0.0127 (0.0066)	−0.0226 (0.0041) ***	0.0308 (0.0066)***	−0.0012 (0.0064)	0.0423 (0.0089)***
Race/ethnicity					
Black or African-American	−0.0014 (0.0046)	−0.0223 (0.0053)***	−0.0900 (0.0049)***	−0.0855 (0.0058)***	−0.1189 (0.0159)***
Hispanic or Latino	−0.0691 (0.0057)***	−0.0437 (0.0064)***	−0.0634 (0.0059)***	−0.0872 (0.0074)***	−0.0826 (0.0156)***
Asians/other	−0.0213 (.0080)**	0.0154 (0.0054)**	−0.0640 (0.0078)***	−0.0066 (0.0081)	0.0079 (0.0082)
Education					
High school or less	−0.01835 (0.0069)***	−0.1507 (0.0112)***	−0.05844 (0.0050)***	−0.6384 (0.0052)***	−0.2004 (0.0142)***
Some college	−0.1105 (0.0067)***	−0.01163 (0.0026)***	−0.3444 (0.0043)***	−0.4382 (0.0038)***	−0.7837 (0.0333)***
Industry					
Ambulatory	0.0287 (0.0042)***	−0.1221 (0.0043)***	−0.02414 (0.0067)***	−0.0775 (0.0042)***	−0.0466 (0.0061)***
Long-term care	−0.2056 (0.0043)***	−0.1881 (0.0038)***	−0.4708 (0.0067)***	−0.0723 (0.0053)***	−0.1419 (0.0496)**
Other/Govt., Educ., Community Org.	−0.1648 (0.0069)***	−0.1795 (0.0052)***	−0.3485 (0.0049)***	−0.1209 (0.0046)***	−0.1499 (0.0115)***
Nativity					
Naturalized citizen	0.0322 (0.0064)***	0.0426 (0.0055)***	−0.0445 (0.0078)***	0.0226 (0.0077)**	−0.0021 (0.0080)
Non-citizen	−0.0482 (0.0078)***	−0.0154 (0.0075)*	−0.2094 (0.0101) ***	−0.0870 (0.0115)***	−0.1277 (0.0134)***
Hours worked					
30 to 34	−0.0646 (0.0053)***	0.0093 (0.0046)*	−0.2802 (0.0093)***	0.0317 (0.0065)***	0.0022 (0.0135)
41 to 50	−0.00569 (0.0056)***	−0.0515 (0.0033)***	0.0096 (0.0040)*	−0.0244 (0.0046)***	−0.0037 (0.0050)
51 or more	−0.3525 (0.0106)***	−0.2615 (0.0073)***	−0.2525 (0.0074)***	−0.03243 (0.0086)***	−0.0871 (0.0098)***
Year					
2004	−0.0500 (0.0316)	0.0965 (0.0190)***	0.0073 (0.0137)	0.0849 (0.0170)***	0.0101 (0.0188)
2008	−0.0584 (0.0293)*	0.1153 (0.0137)***	−0.0469 (0.0117)***	0.0590 (0.0146)***	0.1582 (0.0221)***
2013	−0.0735 (0.0285)*	0.0923 (0.0136)***	−0.0708 (0.0121)***	0.0470 (0.0146)**	0.2287 (0.0187)***
2017	−0.0754 (0.0285)**	0.1109 (0.0132)***	−0.0633 (0.0117)***	0.0287 (0.0145)*	0.2660 (0.0181)***
Full interaction (gender by year)					
2004	0.0657 (0.0327)*	−0.0189 (0.0198)	0.0113 (0.0158)	−0.0415 (0.0190)*	0.1901 (0.0428)***
2008	0.0488 (0.0300)	−0.0255 (0.0145)	0.0169 (0.0133)	−0.0094 (0.0162)	−0.0111 (0.0361)
2013	0.0466 (0.0293)	−0.0095 (0.0144)	0.0125 (0.0137)	0.0003 (0.0162)	0.0524 (0.0368)
2017	0.0762 (0.0292)**	−0.0135 (0.0139)	0.0240 (0.0133)	0.0139 (0.0161)	0.0937 (0.0348)**
State fixed effects	✓	✓	✓	✓	✓
Sample size	78,893	87,835	1,12,080	1,19,223	22,836

Sample is full-time, full-year workers, aged 18–75, except physician and surgeons are ≥ 35 years of age

Source: American Community Survey 2001, 2004, 2008, 2013, 2017

^***^P ≤ 0.001, **P ≤ 0.01, *P ≤ 0.05

Dependent variable is log(hourly wages in real dollars)

In every occupation except physicians, workers younger than 41 made significantly less than employees aged 60–75 (the omitted category). Nurses aged 60–75 years of age earned more than other age groups. Physicians aged 41–59 earned higher median wages than those aged 60–75. African Americans earned less than non-Hispanic Whites (the omitted category) except in healthcare support. Latinos realized wage penalties in every domain. Non-Hispanic Whites made more than Asians/others in healthcare support and direct patient care but earned less in nursing.

Naturalized citizens had higher median wages than native-born workers in healthcare support, healthcare practitioner, and nursing occupations. non-citizens always earned less than native-born workers. Individuals without a Bachelors' Degree (the omitted category) were paid less than those who had completed college.

Jobs located in hospitals (the omitted industry) paid significantly higher median wages when compared to the other three sectors. Long-term care workers in healthcare support, nursing, and direct patient care had 19%, 17%, and 38% lower wages, respectively, than their hospital-based colleagues. Healthcare practitioners and physicians faced their most significant wage penalty in the "other" sector, earning 11% and 14% less than their peers in hospitals.

Those working 30–34 h a week in healthcare support, nurse, and healthcare practitioner jobs had significantly lower wages than workers in the omitted 35–40 h category. Laboring 41–50 h a week lowered wages in the healthcare support, nurse, and healthcare practitioner jobs. And wage penalties were evident for working 51 or more hours across occupations.

Year dummy variable coefficients estimated the difference between the year intercept and the 2001 omitted intercept and accounted for potential changes in wages over time. Table 2 reveals that when compared to 2001, there was a negative wage trend beginning in 2008 for direct patient care and healthcare support laborers. The gender–year interaction term calculated the difference between the effect of gender on median wages in a specific year compared to its impact in 2001. In 2004, women's earnings in healthcare support and physicians increased, while their wages fell in the healthcare practitioner occupation. But the significant 2004 gender interaction coefficient for physicians is suspect as 72.8% of male and 52.9% of female physicians had top-coded earnings that year. Thus, the median wage for all physicians was understated, but the underestimation was more significant for male physicians. In 2017, women in support and healthcare physicians’ made wage gains of 7% and 10%.

### Gender wage gaps by year and occupation

Table 3 provides the predicted median wages for men and women by year and occupation. The log regression coefficients from Table 2 were used to predict wages, and an adjusted gender wage gap was calculated. The wage gap narrowed between 2001 and 2017 in every occupation except for nurse. As a percentage of men's, women's median wages in nursing declined from 95.7% in 2001 to 94.3% in 2017 but still reflected the greater pay parity found in this job. The gender wage gap in the physician occupation appears drastically cut between 2001 and 2004, with women losing ground between 2004 and 2008. However, these findings are likely an artifact of top coding [see Additional file 2].

**Table 3:** Regression-adjusted predicted real median hourly wage by occupation, gender and year

	2001	Annualized		Annualized		Annualized		Annualized		Cumulative
		2001–2004	2004	2004–2008	2008	2008–2013	2013	2013–2017	2017	2001–2017
		% △wages		% △wages		% △wages		% △wages		% △wages
Healthcare support occupations										
Female	$13.54	0.51	$13.75	−0.62	$13.41	−0.35	$13.18	−0.69	$13.55	0.07
Male	$15.65	−1.67	$14.88	−0.19	$14.77	−0.3	$14.55	−0.05	$14.52	−7.22
F/M wage	86.50%		92.40%		90.70%		90.50%		93.30%	
Nurse occupations										
Female	$29.61	2.62	$32.00	0.3	$32.39	−0.14	$32.17	0.36	$32.64	10.23
Male	$30.93	3.29	$34.08	0.47	$34.72	−0.46	$33.93	0.48	$34.58	11.8
F/M wage	95.70%		93.80%		93.20%		94.80%		94.30%	
Direct patient care occupations										
Female	$21.03	0.63	$21.43	−1.21	$20.41	−0.56	$19.84	0.48	$20.22	−3.85
Male	$24.12	0.23	$24.29	−1.34	$23.01	−0.47	$22.47	0.19	$22.64	−6.13
F/M wage	87.10%		88.20%		88.70%		88.20%		89.30%	
Healthcare practitioner and technical occupations										
Female	$21.68	1.45	$22.64	0.15	$22.78	−0.04	$22.73	−0.12	$22.62	4.33
Male	$25.60	2.88	$27.88	−0.65	$27.15	−0.24	$26.83	−0.47	$26.33	2.85
F/M wage	84.60%		81.20%		83.90%		84.70%		85.90%	
Physician and surgeon occupations										
Female	$56.10	6.9	$68.53	−1.32	$64.99	2.71	$74.30	1.99	$80.38	43.2
Male	$72.43	0.33	$73.14	3.78	$84.84	1.43	$91.06	0.92	$94.45	30.4
F/M wage	77.40%		93.60%		76.60%		81.60%		85.10%	

Sample is full-time/full-year workers, aged 18–75, except physicians and surgeons are ≥ 35 years of age

Source: American Community Survey 2001, 2004, 2008, 2013, 2017

In the healthcare support and direct patient care occupations, both men and women were negatively impacted by the great recession, as evidenced in the annualized percentage wage change columns for 2004–2008 and 2008–2013. Additionally, male median wages grew more slowly than women's during 2008–2013 in the nurse, healthcare practitioner, and physician professions. During the recovery years of 2013–2017, both men and women in nurse, direct patient care, and physician jobs realized improved wage growth. Alternatively, all healthcare support and healthcare practitioner employees experienced negative wage growth.

Overall, men in healthcare support registered a negative cumulative wage change, while women experienced wage stagnation. As a group, direct patient care workers experienced cumulative negative wage growth. Women in the healthcare practitioner and physician occupations had higher overall wage growth rates than men, except in nursing.

## Discussion

This study contributes to the international literature documenting the adverse impacts of economic recessions on healthcare workers and existing occupational gender pay disparities [6, 7]. Even as healthcare employment increased between 2001 and 2017, median wage growth was uneven across occupations. Not every occupation was equally disadvantaged by the Great Recessionary shock. Between 2013 and 2017, when wages would be expected to rise as the economic recovery proceeded, only the professional occupations exhibited wage growth for both genders. Gould [30] estimated cumulative, unadjusted, real wage growth at the 50th percentile as 5% for all workers aged 18–64, both full and part-time, between 2000 and 2017. This study's cumulative rates of unadjusted median wage growth were 9.92% for nurses, 5.68% for healthcare practitioners, and 37.6% for physicians (Table 1). These rates are above those reported nationally for the 50th wage percentile, confirming that professionals do better [10, 15, 18].

Wage growth at the 50th percentile of the wage distribution is bifurcated. The less than 1% cumulative growth rate in healthcare support means that these workers earned far less than the 2017 estimated living wage ($16.07) necessary for meeting a family's basic needs [31]. Earnings were limited for men and less-educated workers. Those with only a high school education had wages 17% lower than their college-educated peers.

Consistent with other studies, industry location was a significant determinant of wages, and hospital workers earned more [10, 12]. Between 2001 and 2017, jobs in healthcare support moved to lower-paying sectors. In data not shown but available from the author, by 2017, 47% of healthcare support jobs were in the lower-paying, long-term care, and "other" sectors. There was also an uptick in healthcare support located in ambulatory care [29].

The estimated negative wage growth for direct patient care employees is surprising given the number of skilled and credentialed workers in this category. One explanation lies in the increased percentage of these jobs (56% in 2017) located in the "other" sector. Higher-paid workers such as psychologists and health services managers face a wage "ceiling effect” in the public sector [22]. Were these jobs located in the private sector, they would pay more. Additionally, approximately one-third of the direct patient care category was composed of home/personal care aides in 2017 (data not shown). These jobs are the lowest-paid and least regulated in healthcare [11, 32].

Estimated gender pay gaps for the nurse and physician occupations are comparable to other studies, despite different countries, data sets, and control variables [33–37]. Reductions in the role of observables, such as education and experience, are causing the gender pay gap in professional occupations to close [38]. Since longer work hours were negatively associated with wages, this variable would explain little of the gender wage gap using a decomposition model [38, 39]. By 2017 a higher percentage of women than men worked between 35 and 40 hours a week (Additional file 1: Table S1). More women working these hours means increased earnings across occupations. Collective bargaining coverage for healthcare workers, especially in the public sector, could have reduced wage inequality among men and women over time [40]. Whether the limited labor market mobility of non-White and immigrant men in healthcare support contributed to their wage growth decline requires more investigation [3, 25].

Despite noticeable gains, gender pay disparities in nursing and medicine signal the limitations placed on women's decision-making, promotion, and training opportunities [24, 35, 37]. The substantial pay gap between male and female primary care physicians has been associated with women's additional time spent with patients. More extended face-to-face interactions mean fewer office visits and lower employer revenues, resulting in pay reductions for female physicians [41]. Compensation schemes that discount the value of care services force women to choose between commitment or bargaining for pay [42].

Nurses want respect and collaboration from doctors, the ability to practice at the top of their license, sound referral systems, and adequate resources [24]. But the market has historically devalued skills demanding empathy, dedication, and intrinsic motivation, which form a large part of gendered care service provision [22, 42]. This contributes to the unequal pay between men and women for comparable work in nursing, medicine, and the allied professions.

## Conclusion

Wages are pro-cyclical. The slow or negative median wage growth in all but the physician occupation between 2004–2008 and 2008–2013 confirms that U.S. healthcare wages are not recession-proof, unlike healthcare employment. Workers in the three professional occupations had cumulative rates of wage growth between 2001 and 2017 that were higher than the national rate, confirming these are indeed “good jobs”. Alternatively, wages in both healthcare support and direct patient care exhibited growth rates below the national median.

Women were consistently paid less than men, despite controls for occupation, industry, and human capital. Yet, the gender pay gap narrowed in all professions except nurse. Gender-neutral job evaluation schemes and legal actions for enforcing equal pay must continue to challenge gender pay disparities, especially at the top of the wage distribution where the gap is most significant [33, 38]. Hikes in the federal governments’ reimbursement rates and insurance subsidies raise healthcare workers' income. Increasing the federal minimum wage to $15 or more would overwhelmingly benefit the lowest-paid women and further close the gender pay gap [4].

## Supplementary Information

**Additional file 1: Table S1.** Summary Statistics (2001–2017). Percentage of full-time, full-year workers, aged 18–75, by year, regression variable and gender.

**Additional file 2:** Percent of physicians and surgeons—with top-coded income values. By year and gender. Top-coded physician and surgeon income 2001, 2004, 2010, 2013, 2017.

## Data Availability

The datasets generated during and/or analyzed during the current study are available from the corresponding author on reasonable request.
