# Correction to: Real wage growth in the U.S. health workforce and the narrowing of the gender pay gap

**DOI:** 10.1186/s12960-021-00676-y

**Published:** 2021-11-10

**Authors:** Janis Barry

**Affiliations:** Department of Economics, Fordham University, 113 West 60th Street, New York, NY 10023 USA

## Correction to: Hum Resour Health (2021) 19:105 https://doi.org/10.1186/s12960-021-00647-3

Following the publication of the original article [1], the author identified typos in the article and that the incorrect Additional file 1 was published.

The original article [1] has been corrected (Additional file 1).

## Supplementary Information

**Additional file 1. Table S1.** Summary Statistics (2001–2017).
