# Supplementary material for: Real wage growth in the U.S. health workforce and the narrowing of the gender pay gap
Source: Hum Resour Health. 2021 Aug 28;19:105. doi: 10.1186/s12960-021-00647-3 (PMC8403397; doi:10.1186/s12960-021-00647-3)
Supplement: Supplementary file 2 — Additional file 2: Percent of physicians and surgeons—with top-coded income values. By year and gender. Top-coded physician and surgeon income 2001, 2004, 2010, 2013, 2017. [file 12960_2021_647_MOESM2_ESM.docx]

Additional file 2: Percent of Physicians and Surgeons –with Top-coded Income Values

By Year and Gender. Top-coded Physician and Surgeon Income 2001, 2004, 2010, 2013, 2017

**Percent of Physicians and Surgeons –with Top-coded Income Values**

**By Year and Gender**

|  | **ACS Year** | | | | |
| --- | --- | --- | --- | --- | --- |
| **Gender** | **2001** | **2004** | **2008** | **2013** | **2017** |
| Male | 44.4% | 72.8% | 42.0% | 52.5% | 53.6% |
| Female | 20.5% | 52.9% | 19.1% | 29.4% | 27.8% |

Percentages are based on unweighted counts in the sample data. Sample are aged *35* and older, full-time/full-year with at least 30 hours a week
